# Color Stability of the Bulk-Fill Composite Resins with Different Thickness in Response to Coffee/Water Immersion

**DOI:** 10.1155/2016/7186140

**Published:** 2016-06-14

**Authors:** Sayna Shamszadeh, Seyedeh Mahsa Sheikh-Al-Eslamian, Elham Hasani, Ahmad Najafi Abrandabadi, Narges Panahandeh

**Affiliations:** ^1^Dental Research Center, Research Institute of Dental Sciences, Shahid Beheshti University of Medical Sciences, Tehran 19839 63113, Iran; ^2^Preventive Dentistry Research Centre, Research Institute of Dental Sciences, Shahid Beheshti University of Medical Sciences, Tehran 19839 63113, Iran

## Abstract

We aimed to evaluate the color stability of bulk-fill and conventional composite resin with respect to thickness and storage media. Twenty specimens of a conventional composite resin (6 mm diameter and 2 mm thick) and 40 specimens of the bulk-fill Tetric EvoCeram composite resin at two different thicknesses (6 mm diameter and 2 mm thick or 4 mm thick, *n* = 20) were prepared. The specimens were stored in distilled water during the study period (28 d). Half of the specimens were remained in distilled water and the other half were immersed in coffee solution 20 min/d and kept in distilled water between the cycles. Color changes (Δ*E*) were measured using the CIE *L* ^⁎^ *a* ^⁎^ *b* ^⁎^ color space and a digital imaging system at 1, 7, 14, and 28 days of storage. Data were analyzed using Two-way ANOVA and Tukey's HSD post hoc test (*P* < 0.05). Composite resins showed significant increase in color changes by time (bulk-fill > conventional; *P* < 0.001). Coffee exhibited significantly more staining susceptibility than that of distilled water (*P* < 0.001). There was greater color changes with increasing the increment thickness, which was significant at 14 (*P* < 0.001) and 28 d (*P* < 0.01). Color change of bulk-fill composite resin was greater than that of the conventional one after coffee staining and is also a function of increment thicknesses.

## 1. Introduction

Color stability of composite resin is an important property influencing its clinical longevity, which continues as a challenge inherent to material [1]. Color changes can occur due to various etiologic factors; extrinsic discoloration can occur due to staining in the superficial layer of resin composite, water absorption, surface roughness, smoking, and diet [2, 3]. Intrinsic discoloration could occur as a result of physicomechanical reaction within the material (e.g., the filler and the resin matrix properties) [4].

In oral conditions, composite resins are exposed to different dietary beverages such as coffee which might result in absorption and adsorption of colorants in coffee into the resin surface [5] and consequently undesirable color change. Previous investigations reported the influence of coffee on the color stability of composite resins [6–10].

Generally, it is recommended that the resins should be placed in 2 mm increments to obtain sufficient light transmittance and complete curing of composite resins [11]. Placing the resin in 2 mm increments has some merits including the reduction of the polymerization shrinkage and the voids incorporation between the layers [12]. However, the application of composite resins in an incremental technique and light curing each increment individually is a time-consuming procedure. There is also an increasing possibility of air bubble inclusion or moisture contamination between individual increments of resin composite restorations [13].

To overcome such fallibility, bulk-fill composite resins are introduced. According to the manufactures, these materials can be sufficiently light cured up to 4 mm in a single increment, without influencing the polymerization shrinkage, degree of conversion, or cavity adaptation [14]. In addition, it is claimed that these materials have lower polymerization shrinkage compared to conventional composite resins [15]. Thus, some postoperative problems, such as gap formation and the incidence of recurrent carries, may be diminished. Such merits are probably due to the increased translucency of the bulk-fill composites, which permits greater light transmission. Additionally, the formulation of these materials allows for modulation of the polymerization reaction by applying the stress-relieving monomers, the use of more reactive photoinitiators, and the incorporation of different types of fillers, such as prepolymer particles and fiberglass rod segments [12].

While the manufacturers recommend bulk-filling of these materials up to 4 mm, many clinicians suspect that the depth of cure and mechanical properties might not be suitable for clinical use.

There are few reports on of the effect of increment thickness and the storage media on the discoloration of these bulk-fill resin composites. Since color changes are a concern that affects the population and one of the main reasons for replacing resin-based restorations, this study investigated the effects of the increment thickness and the storage media on the discoloration of one of these bulk-fill resin composites.

## 2. Materials and Methods

### 2.1. Materials

Two A3 shade light cured composite resins, conventional and bulk-fill Tetric EvoCeram, were selected for this study. Characteristics of the materials are presented in Table 1.

### 2.2. Methods

The specimens of conventional (6 mm diameter and 2 mm thickness, *n* = 20) and bulk-fill composite resins at two different thicknesses (6 mm diameter and 2 mm and 4 mm thickness, *n* = 20) were prepared using a polyethylene mold. After applying the composite resin, a Mylar strip was placed and pressed with a glass slide to obtain a flat surface. The glass slide was removed and the specimens were cured for 40 s using a halogen light curing unit (1086.67 mW/cm^2^, Demetron L.C; Kerr Corporation, Orange, CA, USA).

After removing the specimens from the molds, baseline color was measured using a digital image analysis method as described later. Specimens were stored at 37°C in distilled water for 28 d.

Half of the specimens remained in distilled water and the other half were immersed in coffee solution 20 min/d and remained in distilled water in the interval between cycles. To prepare the coffee solution, 25 g of coffee (Taster's Choice, Nestlé USA, Inc., Glendale, CA, USA) was poured in 250 mL of boiling water, and after 10 min of stirring, the solution was filtered through a filter paper.

The color of the specimens was assessed in the Commission International de I'Eclairege *L* ^*∗*^ *a* ^*∗*^ *b* ^*∗*^ (CIELAB) color space using a digital image analysis method. The CIELAB system is a chromatic value color space that measures chroma and value in three coordinates: *L* ^*∗*^, the lightness measured from 0 (black) to 100 (white), *a* ^*∗*^, color in the red (*a* < 0) and green (*a* > 0) axis, and *b* ^*∗*^, color in the blue (*b* < 0) and yellow (*b* > 0) axis.

A setup was designed in a dark room while two 60 w light sources were positioned from the sides (45-degree angle to the samples). At each interval, the specimens were rinsed with distilled water for 5 seconds, blotted dried with tissue paper, and placed on a dark background. 18% grey card was placed next to the specimens to achieve neutral background. The digital images connected with the computer running Adobe Photoshop CS5 (Adobe, San Jose, CA, USA) as color assessment software. The color change (Δ*E*) was calculated using the following equation:

(1) $$\begin{array}{c} \Delta E={\left(\;\Delta L^{2}+\Delta a^{2}+\Delta b^{2}\;\right)}^{1/2}. \end{array}$$

See [16].

### 2.3. Statistical Analysis

The results were statistically analyzed using Repeated Measures Two-way ANOVA with factors including materials (conventional, 2 mm and 4 mm bulk-fill resin composite) and immersion media (coffee and distilled water) for each time interval. For multiple comparisons, Tukey's HSD post hoc test was used to compare different materials (*P* < 0.05) (SPSS v12.0; SPSS Inc., Chicago, IL, USA).

## 3. Results

The mean Δ*E* values of specimens at different thicknesses and media are shown in Table 2. The results of this study showed the significant color changes among all groups (*P* < 0.001). Tukey's HSD test revealed that bulk-fill composite resins showed the significantly greater Δ*E* values compared to those of conventional resins in all time intervals (*P* < 0.001).

Regarding color changes of bulk-fill composite resin at increasing depths, 4 mm thick specimens showed a significant increase in mean Δ*E* values at 14 d (*P* < 0.001) and 28 d (*P* < 0.001). However, the difference was not significant at 1 d and 7 d.

Immersion in coffee and distilled water provided significant color changes. Immersion in coffee solution resulted in greater and significant discoloration over time, when compared to that of water storage (*P* < 0.001).

## 4. Discussion

Color stability of dental restorations is one of the most important characteristics of composite resin materials in terms of longevity [17]. Although there have been several studies on the effect of different beverages on the color stability of resin composites, there is little information regarding the color stability of a new bulk-fill resin composite, which has been introduced for applying in thicker layers.

In the present study the effect of coffee staining on the color stability of bulk-fill (with different thicknesses) and conventional composite resins was evaluated over a period of 28 days. Coffee (20 minutes daily) and distilled water were chosen as the storage media in this study since they mimic the liquids that are constituently in contact with resin composite restorations in the oral environment.

The CIELAB color system was chosen for the color assessment in a current study. This system is a standard method for measuring color differences based on human perception. The Δ*E* value presents relative color differences of dental materials or tooth surfaces before and after an intervention. According to literature, values of Δ*E* < 1 are regarded as not appreciable by the human eye. Values 1 < Δ*E* < 3.3 are considered appreciable by skilled operators but clinically acceptable, whereas values of Δ*E* > 3.3 are considered appreciable by nonskilled persons and are, hence, not clinically acceptable [18]. Therefore, color changes above a value of Δ*E* = 3.3 were considered clinically unacceptable.

In this study significant differences in Δ*E* values were found among materials for both storage media. Coffee staining produced higher color changes in the specimens than those of water storage, which was in line with the previous investigations; Ertaş et al. and Villalta et al. immersed composite resin into the different beverages and reported that coffee showed greater color changes compared to water storage [6, 7]. The present result seems reasonable because when specimens were submitted to coffee staining, discoloration occurred due to the adsorption and absorption of pigments into the organic phase of resin-based materials [19]. Additionally, coffee contains significant amounts of staining agents such as gallic acid, which facilitate staining.

The findings of our study present that water storage can also lead to slight discoloration, slightly perceptible, which is in line with the other investigations. The staining susceptibility of specimens after being immersed in distilled water might be due to their degree of water absorption and the hydrophilic/hydrophobic nature of the resin matrix. Water absorption occurs mainly as direct absorption by the resin matrix [20]. Excessive water absorption can decrease the life of a composite resin by plasticizing and expanding the resin component, causing microcrack formation. As a result, microcracks or interfacial gaps at the interface between the filler and matrix allow stain penetration and discoloration [21]. In addition, discoloration might be due to the differences in the refractive index of filler and matrix which might increase after water absorption [22].

Others factors that have been shown to have a significant impact on the color stability of material are the composition of composite resins and the characteristics of filler particle [23]. In this study the color susceptibility of bulk-fill composite resin was significantly higher than that of conventional composite resin after immersion in storage media; it is claimed that bulk-fill Tetric EvoCeram resins consist of a variety of fillers, prepolymer shrinkage stress reliever, different photoinitiator system, and light sensitivity system. These differences might influence the staining susceptibility [24]. The mechanism of the interaction between the bulk-fill composite resin and the storage media is unknown and requires further investigation.

Altering the resin thickness is one of the other variables that can affect the final appearance of composite restoration. In the present study we tested the different thicknesses (2 and 34 mm) on the color stability of bulk-fill composite resin. We found that bulk-fill materials showed significantly greater discoloration at increasing increment thickness at 14 d and 28 d. This can be explained by the polymerization process of resin-based composites. Depth of cure can be influenced by different factors such as the monomer composition, filler content, and photoinitiator system. Light exposure can lead to causing activation of the photoinitiator which is attenuated by composite absorption and scattering. Thus, depth of cure relies on the material's capacity to transfer light into its depths, as well as on the polymerization kinetics. A previous study showed that, in the case of applying composite resin incrementally, no significant difference was observed in values of depth of cure at different depths. But a bulk-fill technique might result in a greater number of particle/resin matrix interfaces and increased light scattering due to the differences in their refractive indices. Therefore, lesser amount of photons would reach deeper layers of composite resin and consequently a lower depth of cure value would be obtained at the deepest depths. In accordance with these results, Flury et al. reported lower depths of cure of the bulk-fill specimens with 4 mm thickness compared to the values asserted by the manufacturers [25]. In part, differences in the depth of cure, and even the overall degree of conversion, might affect the uncured monomer released and influence the composite discoloration [26]. As a consequence, mechanical properties are deteriorated leading to greater monomer elution which can result in more water absorption and, hence, discoloration.

## 5. Conclusions

Our findings demonstrated that bulk-fill composite resin had greater color susceptibility after immersion in coffee than conventional composites. Considering the increment thickness it can be noted that the discoloration is increased with greater increment thickness. We demonstrated that greater staining susceptibility of thicker specimens might be due to their lower depth of cure when placing bulk-fill materials.

## Figures and Tables

**Table 1:** Composition of the materials.

Materials	Composition	Filler amount, wt%, vol%	Manufacturer
Tetric EvoCeram universal	Bis-GMA, UDMA, Ba-Al-Si glass prepolymer filler (monomer, glass filler, ytterbium trifluoride, and mixed oxide), and prepolymer	55–57	Ivoclar Vivadent (Schaan, Liechtenstein)

Tetric EvoCeram bulk-fill	Dimethacrylates: Bis-GMA, Bis-EMA, UDMA, barium glass, ytterbium trifluoride, mixed oxide, and prepolymer; additives, catalysts, stabilizers, and pigments	81–61	Ivoclar-Vivadent (Schaan, Liechtenstein)

UDMA, urethane dimethacrylate; Bis-GMA, bisphenol A diglycidyl ether dimethacrylate; Ba-Al-Si glass, barium-aluminum-silicate glass.

**Table 2:** Mean (SD) of Δ*E* values at different time intervals and storage media.

Material (thickness)	Time interval	Storage media	
		Water	Coffee
Conventional (2 mm)	Δ*E*_1 d_	2.1 (0.21)^Aa^	2.86 (0.34)^Ab^
	Δ*E*_7 d_	3.26 (0.22)^Aa^	4.97 (0.54)^Ab^
	Δ*E*_14 d_	4.41 (0.20)^Aa^	8.11 (0.46)^Ab^
	Δ*E*_28 d_	5.59 (0.26)^Aa^	11.34 (0.56)^Ab^

Bulk-fill (2 mm)	Δ*E*_1 d_	3.39 (0.36)^Ba^	7.64 (0.58)^Bb^
	Δ*E*_7 d_	4.03 (0.33)^Ba^	11.26 (0.61)^Bb^
	Δ*E*_14 d_	5.14 (0.24)^Ba^	13.84 (0.75)^Bb^
	Δ*E*_28 d_	7.57 (0.34)^Ba^	17.02 (0.86)^Bb^

Bulk-fill (4 mm)	Δ*E*_1 d_	3.12 (0.22)^Ba^	8.00 (0.53)^Bb^
	Δ*E*_7 d_	4.52 (0.24)^Ba^	11.77 (0.44)^Bb^
	Δ*E*_14 d_	5.61 (0.28)^Ca^	17.11 (0.52)^Cb^
	Δ*E*_28 d_	7.50 (0.35)^Ca^	21.31 (0.40)^Cb^

Δ*E*_1 d_,  Δ*E* between 1 day and baseline; Δ*E*_7 d_, Δ*E* between 7 days and baseline; Δ*E*_14 d_, Δ*E* between 14 days and baseline; Δ*E*_28 d_, Δ*E* between 28 days and baseline. Different lowercase letters indicate statistically significant differences between the media (*P* ≤ 0.05). Different uppercase letters indicate statistically significant differences between materials (*P* ≤ 0.05).

## References

[1] Ruyter I. E., Nilner K., Möller B. (1987). Color stability of dental composite resin materials for crown and bridge veneers. *Dental Materials*.

[2] Nasim I., Neelakantan P., Sujeer R., Subbarao C. V. (2010). Color stability of microfilled, microhybrid and nanocomposite resins—an in vitro study. *Journal of Dentistry*.

[3] Gönülol N., Yılmaz F. (2012). The effects of finishing and polishing techniques on surface roughness and color stability of nanocomposites. *Journal of Dentistry*.

[4] Vogel R. I. (1975). Intrinsic and extrinsic discoloration of the dentition. (A literature review). *Journal of Oral Medicine*.

[5] Um C. M., Ruyter I. E. (1991). Staining of resin-based veneering materials with coffee and tea. *Quintessence International*.

[6] Ertaş E., Güler A. U., Yücel A. Ç., Köprülü H., Güler E. (2006). Color stability of resin composites after immersion in different drinks. *Dental Materials Journal*.

[7] Villalta P., Lu H., Okte Z., Garcia-Godoy F., Powers J. M. (2006). Effects of staining and bleaching on color change of dental composite resins. *Journal of Prosthetic Dentistry*.

[8] Khokhar Z. A., Razzoog M. E., Yaman P. (1991). Color stability of restorative resins. *Quintessence International*.

[9] Gross M. D., Moser J. B. (1977). A colorimetric study of coffee and tea staining of four composite resins. *Journal of Oral Rehabilitation*.

[10] Hasani-Tabatabaei M., Yassini E., Moradian S., Elmamooz N. (2009). Color stability of dental composite materials after exposure to staining solutions: a spectrophotometer analysis. *Majallah i Dandanpizishki*.

[11] Pilo R., Oelgiesser D., Cardash H. S. (1999). A survey of output intensity and potential for depth of cure among light-curing units in clinical use. *Journal of Dentistry*.

[12] Bucuta S., Ilie N. (2014). Light transmittance and micro-mechanical properties of bulk fill vs. conventional resin based composites. *Clinical Oral Investigations*.

[13] Tarle Z., Attin T., Marovic D., Andermatt L., Ristic M., Tauböck T. T. (2015). Influence of irradiation time on subsurface degree of conversion and microhardness of high-viscosity bulk-fill resin composites. *Clinical Oral Investigations*.

[14] Ilie N., Bucuta S., Draenert M. (2013). Bulk-fill resin-based composites: an in vitro assessment of their mechanical performance. *Operative Dentistry*.

[15] Czasch P., Ilie N. (2013). In vitro comparison of mechanical properties and degree of cure of bulk fill composites. *Clinical Oral Investigations*.

[16] Jarad F. D., Russell M. D., Moss B. W. (2005). The use of digital imaging for colour matching and communication in restorative dentistry. *British Dental Journal*.

[17] Mundim F. M., Pires-de-Souza F. D. C. P., Garcia L. D. F. R., Consani S. (2010). Colour stability, opacity and cross-link density of composites submitted to accelerated artificial aging. *The European Journal of Prosthodontics and Restorative Dentistry*.

[18] Yap A. U., Sim C. P., Loh W. L., Teo J. H. (1999). Human-eye versus computerized color matching. *Operative Dentistry*.

[19] Van Groeningen G., Jongebloed W., Arends J. (1986). Composite degradation *in vivo*. *Dental Materials*.

[20] Øysæd H., Ruyter I. E. (1986). Water sorption and filler characteristics of composites for use in posterior teeth. *Journal of Dental Research*.

[21] Bagheri R., Tyas M. J., Burrow M. F. (2007). Subsurface degradation of resin-based composites. *Dental Materials*.

[22] Shortcill A. C., Palin W. M., Burtscher P. (2008). Refractive index mismatch and monomer reactivity influence composite curing depth. *Journal of Dental Research*.

[23] Azzopardi N., Moharamzadeh K., Wood D. J., Martin N., van Noort R. (2009). Effect of resin matrix composition on the translucency of experimental dental composite resins. *Dental Materials*.

[24] Orłowski M., Tarczydło B., Chałas R. (2015). Evaluation of marginal integrity of four bulk-fill dental composite materials: in vitro study. *The Scientific World Journal*.

[25] Flury S., Hayoz S., Peutzfeldt A., Hüsler J., Lussi A. (2012). Depth of cure of resin composites: is the ISO 4049 method suitable for bulk fill materials?. *Dental Materials*.

[26] Janda R., Roulet J.-F., Kaminsky M., Steffin G., Latta M. (2004). Color stability of resin matrix restorative materials as a function of the method of light activation. *European Journal of Oral Sciences*.

